# Neoadjuvant chemoimmunotherapy versus chemoradiotherapy for esophageal squamous cell carcinoma: a multicenter cohort study

**DOI:** 10.3389/fimmu.2026.1882240

**Published:** 2026-07-13

**Authors:** Yixiang Zhu, Zan Hou, Yiting Sun, Lei Xu, Yuqin Cao, Lijun Liang, Yongkui Yu, Mei Lan, Ming Fan, Lin Peng, Xuefeng Leng, Chenghao Wang, Linrui Gao, Jian Zheng, Lei Wu, Zefen Xiao, Wei Huang, Ming Wu, Wencheng Zhang, Hecheng Li, Qifeng Wang

**Affiliations:** 1 Department of Radiation Oncology, Sichuan Clinical Research Center for Cancer, Sichuan Cancer Hospital & Institute, Sichuan Cancer Center, University of Electronic Science and Technology of China, Chengdu, China; 2 Department of Thoracic Surgery, Ruijin Hospital, Shanghai Jiao Tong University School of Medicine, Shanghai, China; 3 Departments of Thoracic Surgery, Shanghai Chest Hospital of Shanghai Jiao Tong University School of Medicine, Shanghai, China; 4 Department of Thoracic Surgery, The Second Affiliated Hospital Zhejiang University School of Medicine, Hangzhou, China; 5 Department of Thoracic Surgery, The Affiliated Cancer Hospital of Zhengzhou University & Henan Cancer Hospital, Zhengzhou, China; 6 Department of Thoracic Surgery, Sichuan Clinical Research Center for Cancer, Sichuan Cancer Hospital & Institute, Sichuan Cancer Center, Affiliated Cancer Hospital of University of Electronic Science and Technology of China, Chengdu, China; 7 Department of Radiation Oncology, Tianjin Medical University Cancer Institute & Hospital, National Clinical Research Center for Cancer, Key Laboratory of Cancer Prevention and Therapy, Tianjin’s Clinical Research Center for Cancer, Tianjin, China; 8 Department of Radiation Oncology, National Cancer Center/National Clinical Research Center for Cancer/Cancer Hospital, Chinese Academy of Medical Sciences and Peking Union Medical College, Beijing, China; 9 Department of Radiation Oncology, Shandong Cancer Hospital and Institute, Shandong First Medical University and Shandong Academy of Medical Sciences, Jinan, China

**Keywords:** disease-free survival (DFS), locally advanced esophageal squamous cell carcinoma (LA-ESCC), neoadjuvant chemoimmunotherapy (NCIT), neoadjuvant chemoradiotherapy (NCRT), overall survival (OS)

## Abstract

**Purpose:**

The comparative efficacy of neoadjuvant chemoimmunotherapy (NCIT) vs neoadjuvant chemoradiotherapy (NCRT) for locally advanced esophageal squamous cell carcinoma (LA-ESCC) remains controversial.

**Methods and materials:**

This multicenter retrospective cohort study included patients with LA-ESCC who received NCIT or NCRT followed by esophagectomy across 7 Chinese medical centers between January 2012 and January 2024. The primary outcomes were disease-free survival (DFS) and overall survival (OS). Propensity score matching (PSM) was utilized to balance baseline covariates.

**Results:**

Among 2535 enrolled patients, 1414 received NCIT and 1121 received NCRT. After 1:1 PSM, 1258 patients (629 per group) were evaluated. With a median follow-up of 32.7 months, no significant differences were observed between the NCIT and NCRT groups in DFS (hazard ratio [HR], 1.14; 95% CI, 0.94-1.38; *P* = .20) or OS (HR, 0.98; 95% CI, 0.77-1.25; *P* = .89). The 2-year DFS rates were 68.7% (NCIT) vs 72.0% (NCRT), and 2-year OS rates were 81.3% vs 83.7%, respectively. NCIT showed a trend toward improved distant metastasis-free survival (DMFS) (HR, 0.87; 95% CI, 0.69-1.11; P = .27), whereas NCRT was associated with a trend toward improved locoregional recurrence-free survival (LRFS) (HR, 1.22; 95% CI, 0.96-1.56; P = .11).

**Conclusions:**

NCIT and NCRT demonstrated comparable DFS and OS. These findings suggest that both modalities are valid neoadjuvant strategies, possessing differing strengths in local vs systemic tumor control. These observations await confirmation in prospective trials.

## 1 Introduction

Esophageal cancer poses a significant challenge to public health systems worldwide, ranking as the seventh most common cancer globally and the sixth leading cause of cancer-related mortality (1). In Eastern populations, particularly in China, esophageal squamous cell carcinoma (ESCC) constitutes over 90% of all incident cases (2). The disease is characterized by an exceptionally aggressive biological behavior, early submucosal lymphatic dissemination, and a propensity for widespread locoregional and distant metastasis (3). For more than a decade, neoadjuvant chemoradiotherapy (NCRT) followed by radical esophagectomy has stood as the standard for resectable LA-ESCC^4^. Reported outcomes include a pathological complete response (pCR) rate of 43.2%–49% and a 3-year overall survival (OS) rate of 65.5%–67% (4, 5).

Despite the profound local control afforded by NCRT, the plateauing of 5-year survival rates near 50% and the persistent threat of hematogenous distant metastasis have catalyzed the search for enhanced systemic therapies (6). The advent of immune checkpoint inhibitors (ICIs), specifically monoclonal antibodies targeting the programmed cell death protein 1 (PD-1) pathway, has fundamentally disrupted the therapeutic landscape (7–9). Compared with chemotherapy alone, the combination of ICIs and chemotherapy has significantly improved OS in the first-line treatment of advanced ESCC (10–12). Consequently, researchers have begun exploring whether this combination can enhance pathological response and survival outcomes in patients with LA-ESCC. A recent phase III randomized clinical trial compared the efficacy of neoadjuvant chemotherapy with or without immunotherapy in LA-ESCC, showing that neoadjuvant chemotherapy plus immunotherapy (NCIT) significantly improved the pCR (28.0% vs 4.7%, P < .0001) (13). This suggests that NCIT has emerged as a new neoadjuvant therapeutic strategy for LA-ESCC.

However, this rapid clinical adoption has birthed a highly polarized and contentious debate regarding the comparative effectiveness of NCIT versus the established NCRT standard. Recently, some comparative analyses between NCIT and NCRT have yielded conflicting survival and pathological outcomes, NCIT and NCRT with a similar pCR (22.9%-27.5% vs 25.9%-36.4%, P >.05), and NCIT with a better disease-free survival (DFS) (73.9%-87.4% vs 63.4%-72.8%, P <.05) (14, 15). Based on these findings, some scholar have suggested that radiation therapy may be safely de-escalated or omitted. However, the reporting of a mere 25.9% pCR rate within their NCRT cohort (14), which different from the landmark CROSS and NEOCRTEC5010 trials (pCR: 43.2%-49%, 2-year DFS: 75.8% for NCRT) (4, 5). Consequently, whether NCIT fundamentally surpasses NCRT in optimizing long-term therapeutic outcomes for LA-ESCC remains unresolved. Therefore, we analyzed clinical data from seven tertiary hospitals in China on LA-ESCC, aiming to provide important insights into the optimal neoadjuvant treatment strategy for LA-ESCC.

## 2 Materials and methods

This multicenter retrospective study was conducted across seven medical institutions in China. The study protocol was approved by the Ethics Committee for Medical Research and New Medical Technology of Sichuan Cancer Hospital (SCCSMC-01-2026-048), the Tianjin Medical University Cancer Institute & Hospital, National Clinical Research Center for Cancer (bc20250579), and Ruijin Hospital, Shanghai Jiao Tong University School of Medicine (2025–768). A waiver of informed consent was granted for all participating centers due to the use of anonymized retrospective data. The reporting of this study adhered to the STROBE (Strengthening the Reporting of Observational Studies in Epidemiology) guidelines.

**Figure 1:**
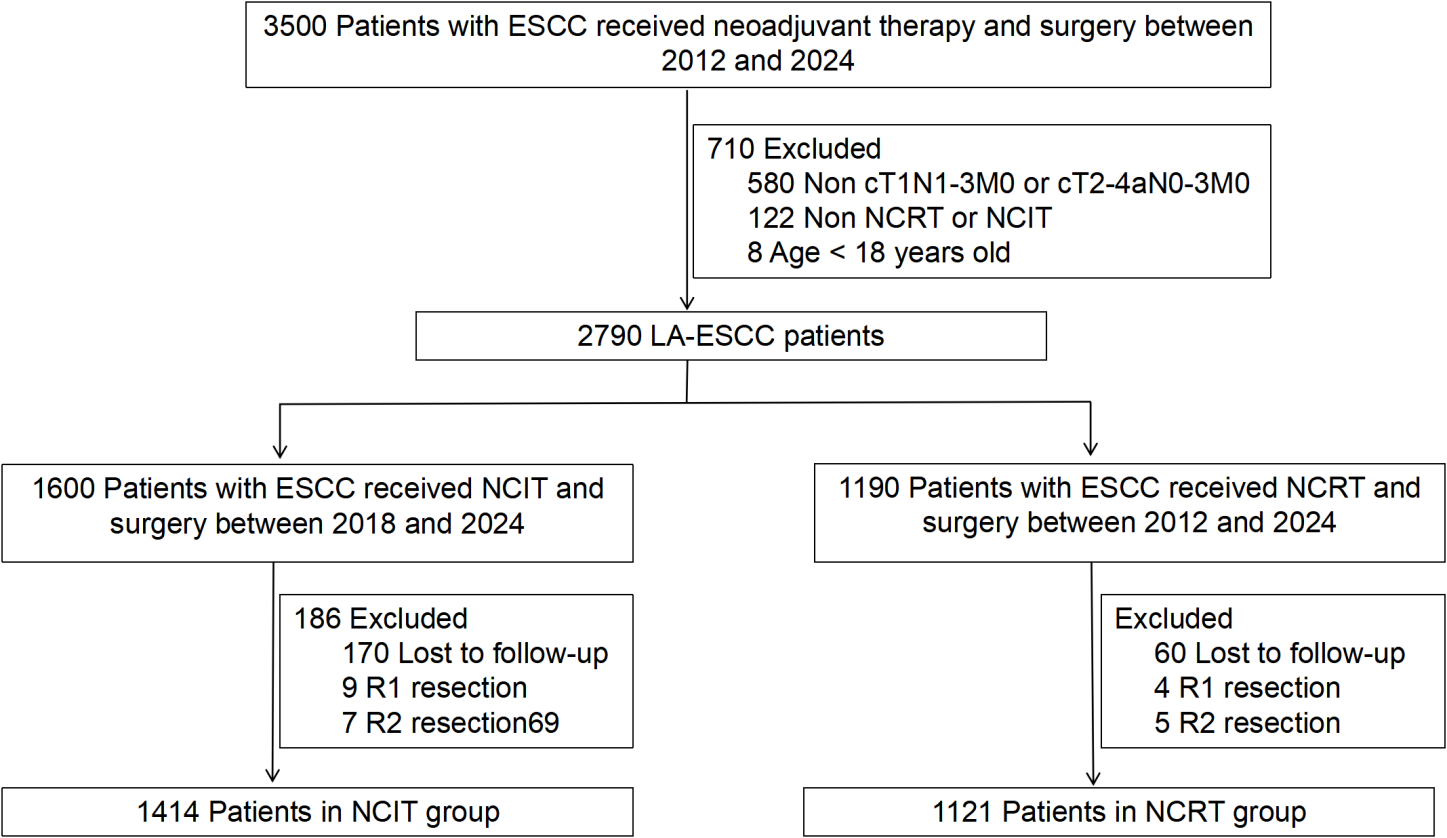
Study enrollment flowchart. ESCC, esophageal squamous cell carcinoma; NCRT, neoadjuvant chemoradiotherapy; NCIT, neoadjuvant immunotherapy plus chemotherapy.

Patients with pathologically confirmed LA-ESCC were enrolled from participating centers between January 2012 and October 2024. Inclusion criteria were: (1) clinical staging based on the 8th edition of the AJCC TNM staging system, classified as cT1N1–3M0 or cT2–4aN0–3M0; (2) age ≥18 years; (3) adequate hematologic, renal, hepatic, cardiac, and pulmonary function; and (4) treatment with NCRT or NCIT. Exclusion criteria included: (1) with other malignant tumors (excluding cured skin cancer); (2) non squamous cell carcinoma such as adenocarcinoma and small cell carcinoma; (3) receipt of other preoperative therapies; (4) absence of surgical resection; and (5) loss to follow-up. The patient screening process is summarized in **Figure 1**.

In the NCIT group, patients received 2–3 cycles of PD-1 inhibitors (including pembrolizumab, nivolumab, camrelizumab, sintilimab, toripalimab, or tislelizumab administered every three weeks) in combination with chemotherapy. For those undergoing NCRT, neoadjuvant radiotherapy was delivered at a total dose of 40.0–41.4 Gy in 1.8–2.0 Gy fractions, five days per week. Both groups received 2–3 cycles of platinum-based doublet chemotherapy, consisting of a platinum agent (cisplatin, carboplatin, or nedaplatin) paired with either paclitaxel or fluorouracil. Both groups allow adjuvant therapy.

Pathologic TNM staging was performed according to the 8th AJCC criteria. Histopathologic specimens were independently evaluated by two experienced pathologists, with any discrepancies resolved through multidisciplinary team (MDT) discussions. R0 resection was defined as complete tumor removal with negative surgical margins (distal, proximal, and circumferential). pCR was defined as the absence of viable tumor cells in both the primary site and all resected lymph nodes. Tumor response to neoadjuvant therapy was assessed using the Chirieac Tumor Regression Grade (TRG) system, which categorizes responses into four grades: TRG 0 (no residual tumor), TRG 1 (<10% residual tumor), TRG 2 (10–50% residual tumor), and TRG 3 (>50% residual tumor). Major pathologic response (MPR) was defined as TRG 0 or 1, indicating <10% residual tumor in the primary site, irrespective of nodal status.

DFS was defined as the time from resection treatment to disease progression or death from any cause, with patients who did not progress or die censored at the last follow-up. OS was defined as the time from the start of the resection treatment to death from any cause, with participants who did not die censored at the time of the last follow-up. Locoregional recurrence-free survival (LRFS) was defined as the time from resection treatment to locoregional recurrence or death from any cause, with patients who did not progress or die censored at the last follow-up. Distant metastasis-free survival (DMFS) was defined as the time from resection treatment to distant metastasis or death from any cause, with patients who did not progress or die censored at the last follow-up. The follow-up cutoff was January 31, 2025.

Patient follow-up began after the resection and occurred every 2–4 months for the first two years, every 6 months for years three to five, and annually afterward. Follow-up included examinations such as enhanced computed tomography (CT) scans of the chest and upper abdomen. The efficacy of antitumor therapy was evaluated independently by two radiation oncologists according to the Response Evaluation Criteria in Solid Tumors, version 1.1 (RECIST 1.1), with any disputes resolved by negotiation. We compared the DFS, OS, LRFS and DMFS of patients with different treatment regimens. Age, sex, smoking history, alcohol history, ECOG Performance Status (PS) score, tumor location, body mass index (BMI), cTN stage, yTN stage, pCR status, MPR status, and adjuvant therapy status were included in the univariate and multivariate analyses.

Categorical variables are presented as counts and percentages. Survival curves for DFS, OS, LRFS and DMFS were generated using the Kaplan–Meier method and compared with the log-rank test. Hazard ratios (HR) and 95% confidence intervals (CI) were estimated using Cox proportional hazards models. Independent prognostic factors for DFS and OS were identified through stepwise univariate and multivariate Cox regression analyses for all patients. Variables with p < 0.1 in univariate analysis were included in the multivariate model. To rigorously address potential selection bias inherent in the non-randomized design, we pre-specified propensity score matching (PSM) as key sensitivity analyses. PSM was applied to balance the baseline characteristics between the two groups. Standardized mean differences (SMD) were used to assess the balance before and after weighting, with an SMD < 0.20 considered indicative of good balance. All statistical analyses were performed using R version 4.2.3, with two-tailed P < 0.05 considered statistically significant.

## 3 Results

A total of 2535 patients were included in this study (2144 male [84.6%], 1531 patients ≥ 65 years old [60.4%]), with 1414 patients receiving NCIT and 1121 patients receiving NCRT. Baseline characteristics are detailed in **Table 1**. Significant differences between the two groups were observed in terms of KPS, clinical T category, clinical N category, location, BMI, and adjuvant therapy status. The NCIT group generally had better PS (0, 1176 patients [83.2%] vs 634 patients [56.6%]), higher BMI (> 18, 1342 patients [94.9%] vs 958 patients [85.5%]), more advanced clinical T stages (cT3-4, 1158 patients [81.9%] vs 733 patients [65.4%]), earlier clinical N stages (N0, 1264 patients [18.7%] vs 128 patients [11.4%]), higher proportion of adjuvant therapy (yes, 790 patients [55.9%] vs 182 patients [16.2%]). To address potential imbalances, PSM was performed. After PSM, there were 629 patients in each group. SMDs of less than 0.10 indicated a well-balanced comparison of baseline characteristics between the groups, as shown in **Table 1**.

**Table 1:** Patient characteristics of patients between the NCRT and NCIT groups before and after propensity score matching.

Characteristic	Before propensity score matching				After propensity score matching			
	NCIT (n=1414)	NCRT (n=1121)	*P* value	SMD	NCIT (n=629)	NCRT (n=629)	*P* value	SMD
Sex								
Male	1203 (85.1)	941 (83.9)	.465	.031	545 (86.6)	549 (87.3)	.802	.019
Female	211 (14.9)	180 (16.1)			84 (13.4)	80 (12.7)		
Age								
<65	829 (58.6)	702 (62.6)	.045	.082	388 (61.7)	370 (58.8)	.327	.059
≥65	585 (41.4)	419 (37.4)			241 (38.3)	259 (41.2)		
Smoking history								
No	471 (33.3)	358 (31.9)	.490	.029	196 (31.2)	197 (31.3)	1.000	.003
Yes	943 (66.7)	763 (68.1)			433 (68.8)	432 (68.7)		
Alcohol history								
No	561 (39.7)	392 (35.0)	.017	.097	256 (40.7)	237 (37.7)	.299	.062
Yes	853 (60.3)	729 (65.0)			373 (59.3)	392 (62.3)		
ECOG PS								
0	1176 (83.2)	634 (56.6)	<.001	.606	437 (69.5)	426 (67.7)	.544	.038
1	238 (16.8)	487 (43.4)			192 (30.5)	203 (32.3)		
cT stage								
1	81 (5.7)	28 (2.5)	<.001	.659	31 (4.9)	26 (4.1)	.294	.109
2	175 (12.4)	360 (32.1)			150 (23.8)	125 (19.9)		
3	1050 (74.3)	537 (47.9)			373 (59.3)	396 (63.0)		
4	108 (7.6)	196 (17.5)			75 (11.9)	82 (13.0)		
cN stage								
0	264 (18.7)	128 (11.4)	<.001	.229	107 (17.0)	96 (15.3)	.587	.078
1	588 (41.6)	474 (42.3)			250 (39.7)	261 (41.5)		
2	451 (31.9)	441 (39.3)			209 (33.2)	219 (34.8)		
3	111 (7.9)	78 (7.0)			63 (10.0)	53 (8.4)		
Tumor location								
Proximal third	130 (9.2)	116 (10.3)	.007	.127	67 (10.7)	54 (8.6)	.396	.077
Middle third	646 (45.7)	442 (39.4)			242 (38.5)	257 (40.9)		
Distal third	638 (45.1)	563 (50.2)			320 (50.9)	318 (50.6)		
Body mass index								
<18	72 (5.1)	163 (14.5)	<.001	.326	58 (9.2)	67 (10.7)	.424	.074
18-24	900 (63.6)	619 (55.2)			368 (58.5)	378 (60.1)		
>24	442 (31.3)	339 (30.2)			203 (32.3)	184 (29.3)		
Adjuvant therapy								
No	624 (44.1)	939 (83.8)	<.001	.906	256 (40.7)	536 (85.2)	<.001	1.039
Yes	790 (55.9)	182 (16.2)			373 (59.3)	93 (14.8)		

NCIT, neoadjuvant chemotherapy plus immunotherapy; NCRT, neoadjuvant chemoradiotherapy.

Before PSM, NCRT showed superior pathological downstaging compared to NCIT: pathological T0 [45.9% (515 of 1121) vs 25.5% (360 of 1414), P <.001], pathological N0 [70.7% (793 of 1121) vs 54.1% (765 of 1414), P <.001], pCR [37.9% (425 of 1121) vs 20.4% (289 of 1414), P <.001], and MPR [44.6% (500 of 1121) vs 36.7% (519 of 1414), P <.001] (**Table 2**). After PSM, these differences remained significant: pathological T0 [48.3% (304 of 629) vs 25.4% (160 of 629), P <.001], pathological N0 [72.5% (456 of 629) vs 53.1% (334 of 629), P <.001], pCR [40.2% (253 of 629) vs 21.6% (136 of 629), P <.001], and MPR [48.0% (302 of 629) vs 36.1% (229 of 629), P <.001] (**Table 2**).

**Table 2:** Pathological outcomes of patients between the NCIT and NCRT groups before and after propensity score matching.

Variable		Before propensity score matching			After propensity score matching		
		NCIT (n=1414)	NCRT (n=1121)	*P* value	NCIT (n=629)	NCRT (n=629)	*P* value
ypT stage							
	0	360 (25.5)	515 (45.9)	<.001	160 (25.4)	304 (48.3)	<.001
	1	270 (19.1)	115 (10.3)		103 (16.4)	53 (8.4)	
	2	273 (19.3)	201 (17.9)		150 (23.8)	120 (19.1)	
	3	478 (33.8)	267 (23.8)		205 (32.6)	142 (22.6)	
	4	33 (2.3)	23 (2.1)		11 (1.7)	10 (1.6)	
ypN stage							
	0	765 (54.1)	793 (70.7)	<.001	334 (53.1)	456 (72.5)	<.001
	1	349 (24.7)	233 (20.8)		146 (23.2)	121 (19.2)	
	2	215 (15.2)	67 (6.0)		100 (15.9)	41 (6.5)	
	3	85 (6.0)	28 (2.5)		49 (7.8)	11 (1.7)	
pCR							
	No	1125 (79.6)	696 (62.1)	<.001	493 (78.4)	376 (59.8)	<.001
	Yes	289 (20.4)	425 (37.9)		136 (21.6)	253 (40.2)	
MPR							
	No	895 (63.3)	621 (55.4)	<.001	400 (63.6)	327 (52.0)	<.001
	Yes	519 (36.7)	500 (44.6)		229 (36.4)	302 (48.0)	

**Figure 2:**
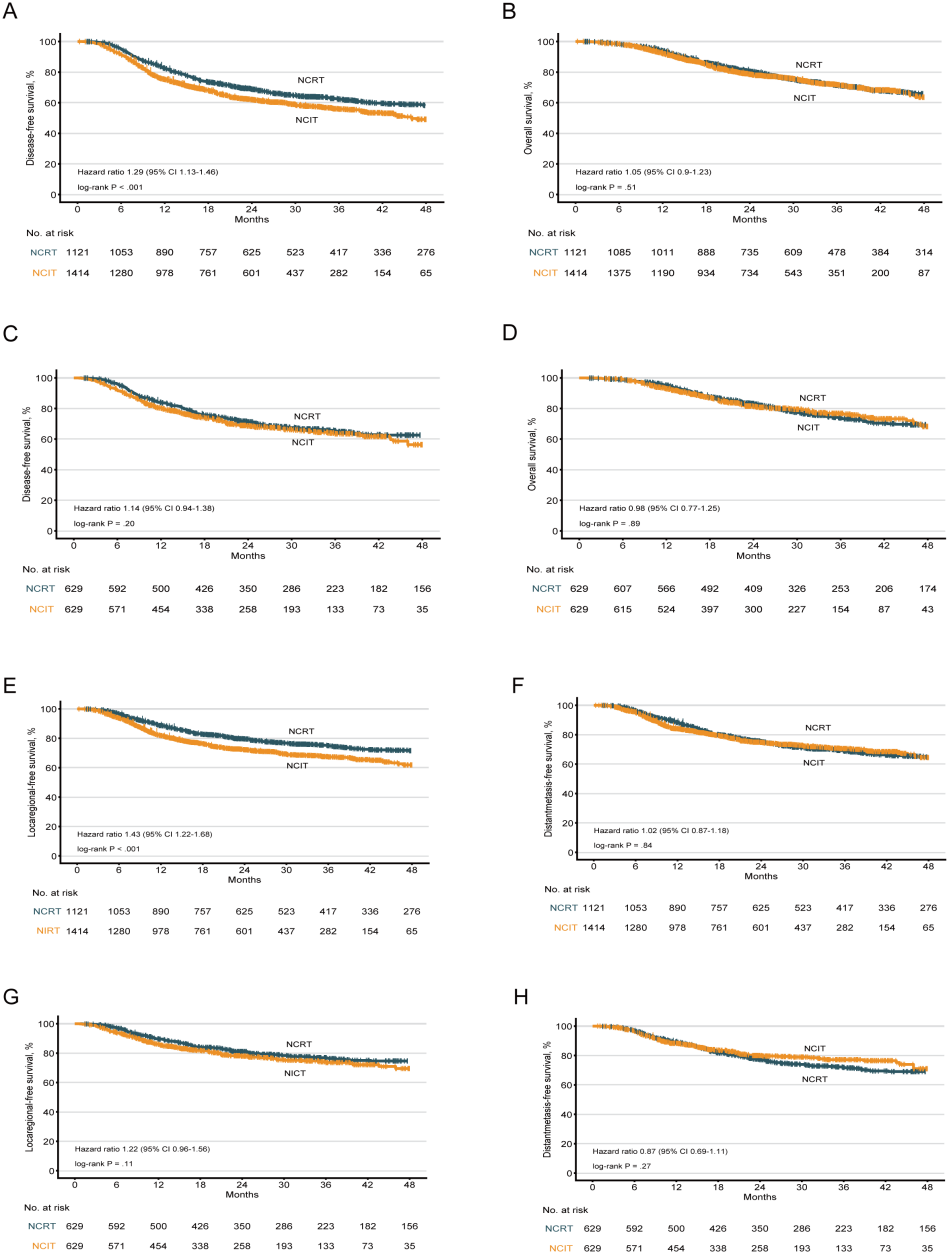
Kaplan-Meier curve of patients receiving NCIT and NCRT. Pre-PSM patient DFS **(A)** and OS **(B)** curves; post-PSM patient DFS **(C)** and OS **(D)** curves. Pre-PSM patient LRFS **(E)** and DMFS **(F)** curves; post-PSM patient LRFS **(G)** and DMFS **(H)** curves. NCIT, neoadjuvant chemotherapy plus immunotherapy; NCRT, neoadjuvant chemoradiotherapy; LRFS, locoregional recurrence-free survival; DMFS, distant metastasis-free survival.

Before PSM, NCRT demonstrated significantly improved 2-year DFS (68.9% vs 62.0%, HR 1.29, 95% CI 1.13–1.46, P <.001) but no difference in OS (85.2% vs 78.5%, HR 1.05, 95% CI 0.90–1.23, P = .51) (**Figures 2A, B**). After PSM, the DFS advantage was attenuated and became non-significant (72.0% vs 68.7%, HR 1.14, 95% CI 0.94–1.38, P = .20), while OS remained comparable (83.7% vs 81.3%, HR 0.98, 95% CI 0.77–1.25, P = .89) (**Figures 2C, D**).

Based on the status of adjuvant therapy, survival differences between NCRT and NCIT were further compared (baseline data are shown in **Supplementary Tables 1**, **2**). Among patients who received adjuvant therapy, NCIT demonstrated similar DFS to NCRT, but exhibited a trend toward better OS than NCRT both before (HR 0.77, 95% CI 0.58–1.01, P = .06) and after PSM (HR 0.64, 95% CI 0.37–1.11, P = .11) (**Supplementary Figure 3**). In patients without adjuvant therapy, NCIT was associated with poorer DFS (HR 1.27, 95% CI 1.01–1.51, P = .01), whereas no difference in OS was observed before PSM (**Supplementary Figure 3**). After PSM, there were no significant differences in DFS or OS between NCIT and NCRT (**Supplementary Figure 3**).

Multivariable analysis identified BMI, ypT stage, ypN stage, and MPR status as independent predictors of DFS, with higher BMI and MPR positivity associated with better outcomes. For OS, BMI, ypT/ypN stages, MPR status, and adjuvant therapy were independent factors, with higher BMI, MPR positivity, and adjuvant therapy conferring survival benefits, while advanced ypT3–4 and ypN+ stages predicted worse outcomes. Neoadjuvant therapy schedule and pCR status were not independently associated with either endpoint (**Table 3**).

**Table 3:** Univariable and multivariable cox proportional hazard analysis evaluating disease-free survival (DFS) and overall survival (OS).

Variable	Disease-free survival				Overall survival			
	Univariate analysis	p value	Multivariate analysis	p value	Univariate analysis	p value	Multivariate analysis	p value
	HR (95% CI)		HR (95% CI)		HR (95% CI)		HR (95% CI)	
Treatment								
NCIT	Reference		Reference		Reference			
NCRT	0.78 (0.69 - 0.88)	< 0.001	0.90 (0.78 - 1.05)	0.169	0.95 (0.81 - 1.11)	0.513		
Sex								
Male	Reference		Reference		Reference		Reference	
Female	0.72 (0.60 - 0.87)	< 0.001	0.91 (0.74 - 1.12)	0.351	0.62 (0.49 - 0.79)	< 0.001	0.81 (0.62 - 1.06)	0.132
Age								
<65	Reference		Reference		Reference			
≥65	0.90 (0.79 - 1.02)	0.095	1.02 (0.90 - 1.17)	0.743	1.05 (0.90 - 1.23)	0.559		
Smoke								
No	Reference		Reference		Reference		Reference	
Yes	1.271 (1.11 - 1.46)	< 0.001	1.08 (0.90 - 1.30)	0.406	1.40 (1.17 - 1.66)	< 0.001	1.13 (0.90 - 1.42)	0.301
Drink								
No	Reference		Reference		Reference		Reference	
Yes	1.26 (1.10 - 1.44)	< 0.001	1.00 (0.84 - 1.19)	0.986	1.35 (1.14 - 1.59)	< 0.001	0.99 (0.80 - 1.23)	0.934
KPS								
0	Reference				Reference			
1	0.99 (0.86 - 1.13)	0.846			1.129 (0.96 - 1.33)	0.142		
Body mass index								
<18	Reference		Reference		Reference		Reference	
18-24	0.64 (0.53 - 0.77)	< 0.001	0.649 (0.534 - 0.789)	< 0.001	0.53 (0.43 - 0.66)	< 0.001	0.57 (0.46 - 0.71)	< 0.001
>24	0.61 (0.50 - 0.75)	< 0.001	0.63 (0.51 - 0.77)	< 0.001	0.48 (0.38 - 0.61)	< 0.001	0.52 (0.41 - 0.67)	< 0.001
Tumor location								
Proximal third	Reference				Reference			
Middle third	0.91 (0.74 - 1.13)	0.388			1.06 (0.81 - 1.39)	0.692		
Distal third	0.92 (0.74 - 1.13)	0.413			1.15 (0.88 - 1.51)	0.298		
cT stage								
1	Reference				Reference			
2	0.83 (0.61 - 1.14)	0.257			1.24 (0.79 - 1.95)	0.351		
3	0.82 (0.61 - 1.10)	0.182			1.27 (0.83 - 1.95)	0.276		
4	0.80 (0.58 - 1.12)	0.194			1.39 (0.88 - 2.20)	0.159		
cN stage								
0	Reference		Reference		Reference		Reference	
1	1.32 (1.07 - 1.63)	0.009	1.05 (0.85 - 1.30)	0.671	1.43 (1.10 - 1.86)	0.008	1.11 (0.85 - 1.45)	0.465
2	1.48 (1.20 - 1.83)	< 0.001	1.04 (0.83 - 1.31)	0.720	1.55 (1.19 - 2.02)	0.001	1.04 (0.79 - 1.38)	0.767
3	2.37 (1.82 - 3.08)	< 0.001	1.08 (0.80 - 1.46)	0.604	2.62 (1.90 - 3.63)	< 0.001	1.13 (0.79 - 1.61)	0.510
ypT stage								
0	Reference		Reference		Reference		Reference	
1	1.59 (1.28 - 1.97)	< 0.001	1.38 (1.00 - 1.89)	0.048	1.39 (1.06 - 1.84)	0.018	1.21 (0.82 - 1.80)	0.336
2	1.90 (1.56 - 2.31)	< 0.001	1.21 (0.91 - 1.62)	0.193	1.82 (1.42 - 2.32)	< 0.001	1.18 (0.82 - 1.69)	0.380
3	3.10 (2.63 - 3.66)	< 0.001	1.77 (1.35 - 2.32)	< 0.001	3.25 (2.65 - 3.97)	< 0.001	1.93 (1.38 - 2.69)	< 0.001
4	4.54 (3.25 - 6.35)	< 0.001	2.42 (1.61 - 3.62)	< 0.001	5.33 (3.71 - 7.66)	< 0.001	2.90 (1.84 - 4.57)	< 0.001
ypN stage								
0	Reference		Reference		Reference		Reference	
1	2.28 (1.97 - 2.65)	< 0.001	1.86 (1.57 - 2.21)	< 0.001	2.20 (1.84 - 2.64)	< 0.001	1.80 (1.46 - 2.21)	< 0.001
2	2.98 (2.49 - 3.55)	< 0.001	2.14 (1.74 - 2.62)	< 0.001	2.75 (2.21 - 3.42)	< 0.001	2.07 (1.61 - 2.65)	< 0.001
3	5.43 (4.33 - 6.822)	< 0.001	3.61 (2.75 - 4.76)	< 0.001	5.83 (4.49 - 7.56)	< 0.001	3.97 (2.91 - 5.40)	< 0.001
pCR								
No	Reference		Reference		Reference		Reference	
Yes	0.35 (0.30 - 0.42)	< 0.001	1.10 (0.75 - 1.62)	0.634	0.35 (0.29 - 0.44)	< 0.001	1.08 (0.66 - 1.75)	0.770
MPR								
No	Reference		Reference		Reference		Reference	
Yes	0.36 (0.31 - 0.42)	< 0.001	0.67 (0.51 - 0.86)	0.002	0.34 (0.29 - 0.41)	< 0.001	0.69 (0.50 - 0.97)	0.032
Adjtherapy								
No	Reference		Reference		Reference		Reference	
Yes	1.39 (1.22 - 1.57)	< 0.001	0.93 (0.81 - 1.08)	0.346	1.20 (1.03 - 1.40)	0.022	0.85 (0.72 - 1.00)	0.047

NCIT, neoadjuvant chemotherapy plus immunotherapy; NCRT, neoadjuvant chemoradiotherapy.

Following PSM, exploratory subgroup analyses revealed that the DFS benefit of NCRT compared to NCIT was evident in patients aged ≥65, those who abstain from drinking and smoking, individuals with an ECOG performance status of 1, a BMI between 18 and 24, and those with clinical N3 stage (**Supplementary Figure 4**). Regarding OS, the advantage of NCRT over NCIT was observed exclusively in patients with an ECOG performance status of 1 (**Supplementary Figure 5**). Furthermore, patients who were female, non-smoking, non-drinking, had an ECOG performance status of 1, a BMI between 18 and 24, and a clinical N2-N3 stage appeared to benefit more from NCRT in terms of LRFS (**Supplementary Figure 6**). Conversely, patients who were female, younger than 65, had an ECOG performance status of 0, and a BMI of 24 or higher seemed to derive greater benefits from NCIT in terms of DMFS (**Supplementary Figure 7**).

Before PSM, NCRT showed lower local failure rates [8.4% (95 of 1121) vs 15.6% (221 of 1414)] but similar distant metastasis rates [15% (169 of 1121) vs 12.3% (175 of 1414)] (**Supplementary Table 3**). The 2-year LRFS and DMFS rates were 79.6% vs 72.2% and 77.1% vs 74.8%, respectively (**Figures 2E, F**). After PSM, local failure remained lower in the NCRT group [7.7% (49 of 629) vs 13.3% (84 of 629)], while distant metastasis rates converged [13.1% (88 of 629) vs 10.5% (66 of 629)]. The 2-year LRFS and DMFS rates were 81.7% vs 77.9% and 79.9% vs 80%, respectively (**Figures 2G,H**). NCRT showed a trend toward better control of local recurrence (LRFS: HR 1.22, 95% CI 0.96–1.56, P = .11) and NCIT showed a trend toward better control of distant metastasis (DMFS: HR 0.87, 95% CI 0.69–1.11, P = .27), though neither reached statistical significance.

## 4 Discussion

To our knowledge, this multicenter retrospective cohort represents the largest sample size to compare the efficacy of NCIT and NCRT in LA-ESCC. It found that NCIT and conventional NCRT yielded comparable DFS and OS outcomes. While NCRT provided markedly superior rates of pCR and MPR—this pronounced pathological advantage did not translate into an overarching survival benefit. NCRT showed a propensity for superior locoregional control compared to NCIT, whereas NCIT demonstrated a potential advantage in mitigating distant metastatic events.

In this study, the most notable finding was a comparable DFS (2-year DFS, 68.7% vs 72.0%) and OS (2-year OS, 81.3% vs 83.7%) between NCIT and NCRT. The 2-year DFS rate of 72.0% within our PSM-adjusted NCRT cohort was closely in the NEOCRTEC 5010 trial (75.8% 2-year DFS) (5), and REVO trial (81.6% 2-year OS) (12). The 2-year OS rates were 81.3% in the NCIT group and 83.7% in the NCRT group, which align with recent reports (ranging from 86.3% to 86.8% for NCIT and 80.2% to 81.6% for NCRT) (16, 17). The 2-year DFS rate in the NCIT group was 68.7%, which is consistent with the rate reported by Guo et al. (14). In contrast, the NCRT group in the same study showed a lower 2-year DFS rate of 63.4% and a 2-year OS rate of 71.3% after PSM. Compared to Guo et al. Study (14), our multicenter cohort included more populations and had a higher proportion of patients presenting with advanced nodal disease (cN2-3). Exploratory subgroup analyses indicated that patients with clinical N2–N3 stage seemed to experience enhanced benefits from NCRT regarding LRFS. The local control attained with concurrent radiotherapy in our NCRT cohort underscores its advantageous role in the management of high-burden locoregional disease—an advantage potentially underappreciated in cohorts with lower baseline nodal staging. The above finding are derived from subgroup analyses and warrant further validation through prospective randomized controlled trials.

In addition, the NCRT group demonstrated substantially higher pCR rates (40.2% vs. 21.6%) compared to the NCIT group. The pCR rate of 40.2% within our PSM-adjusted NCRT cohort closely mirror the robust pathological responses reported in the definitive CROSS (49% pCR) (4), and SCIENCE trial (47.3% pCR) (18). Conversely, Guo et al. (13) reported a substantially lower pCR rate in their NCRT arm (25.9%), comparable to the rate in the NCIT group (22.9%). In a recent phase II study, the NCRT group achieved a pCR rate of 46.2% versus 37.8% with NCIT in the surgery set (16). The pCR rate in the NCRT group was similar to our study, whereas the pCR rate in the NCIT group was higher than our study. In the previous studies, the pCR rates were 15.4%-35.3% in the NCIT group (8, 18–20). One likely factor contributing to the high pCR rate in the phase II trial is the incorporation of nab-paclitaxel in the chemotherapy backbone. Compared with conventional paclitaxel, nab-paclitaxel combined with immunotherapy and platinum-based chemotherapy has demonstrated greater efficacy, leading to higher rate of pCR (21).

Previous studies have indicated that pCR after neoadjuvant treatment had a favorable prognosis (22, 23), however, the relationship between pCR and survival outcomes remains unclear. Although NCRT demonstrated markedly superior pCR and MPR rates, this significant pathological response did not convert to survival advantage in our study. This dissociation between pathological response and survival outcomes represents a crucial finding that warrants careful interpretation. Several factors may explain this apparent paradox. First, the administration pattern of adjuvant therapy introduces a critical confounding dynamic. Within our cohort, a significantly higher proportion of patients in the NCIT group proceeded to receive adjuvant systemic therapy (55.9% vs 16.2%). Consistent with emerging clinical evidence indicating that adjuvant immunotherapy substantially benefits patients with residual disease post-neoadjuvant chemoimmunotherapy (24, 25), this post-surgical intervention likely reduced the survival disadvantage that would otherwise be anticipated from the comparatively lower pCR rates in the NCIT group. Our multivariable analysis corroborates this hypothesis, identifying the receipt of adjuvant therapy as potent, independent predictor of improved survival. Second, the mechanisms of action differ substantially between modalities. While NCRT achieves excellent local tumor control—as reflected in favorable trends in LRFS—It may also trigger immunosuppressive effects that impair long-term immune activity (26). In contrast, NCIT may offer superior systemic immune activation and the establishment of immune memory (27). This immune memory may be more effective at clearing circulating tumor cells and distant micrometastases (as suggested by better distant DMFS trends), and ultimately lower postoperative failure rates (27). Thus, NCIT may offset the immediate local advantages provided by NCRT, thereby conferring a long-term survival benefit. Third, there is cross-over in treatment. In real-world settings, patients in the NCRT group who develop distant metastases may subsequently receive immunotherapy, whereas those in the NCIT group who experience local recurrence or oligometastases may undergo radiotherapy. This crossover treatment strategy could further confound the impact of the initial neoadjuvant modality on eventual survival. Therefore, the pCR rate may not serve as an equivalent surrogate endpoint across different neoadjuvant treatment approaches.

There were several critical limitations in our study. First, the retrospective, observational architecture of the study renders it inherently susceptible to selection bias and unmeasured confounding. Although PSM effectively balanced observed clinical covariates—such as TNM staging and performance status—unmeasured variables, including subtle differences in patient frailty, underlying tumor genomic profiles, and institution-specific surgical expertise, could not be completely neutralized. Second, the substantial imbalance in the receipt of adjuvant therapy between the two study arms complicates the isolation of the pure neoadjuvant effect, accurately reflecting the complex heterogeneity of real-world oncological practice during the evolving study period. Third, this dataset did not systematically capture comprehensive toxicity profiles or specific adverse events (AEs), thereby lacking in a definitive, head-to-head comparison regarding the safety, tolerability, and health-related quality of life associated with the two competing regimens. Fourth, some findings are derived from subgroup analyses and warrant further validation through prospective randomized controlled trials. Finally, while PSM was necessary to ensure baseline comparability, the resulting reduction in the overall cohort size may have constrained the statistical power required to detect smaller, yet potentially clinically meaningful, differences in long-term recurrence patterns.

## 5 Conclusions

In conclusion, this multicenter cohort study demonstrates that both NCIT and NCRT represent valid, effective neoadjuvant strategies for LA-ESCC, yielding comparable DFS and OS. NCRT provides superior pathological downstaging and trend toward better control of local recurrence. Conversely, NCIT offers equivalent OS, an outcome potentially driven by enhanced systemic suppression of distant micrometastases and the subsequent synergistic integration of adjuvant therapy. Consequently, treatment selection for LA-ESCC should evolve beyond a uniform paradigm toward a highly personalized approach, meticulously weighing individual patient risk factors for locoregional failure versus distant metastasis. Prospective randomized clinical trials (28, 29) featuring rigorously standardized neoadjuvant adjuvant treatment protocols are urgently required to definitively establish optimal therapeutic regimen and validate these real-world observations.

## Data Availability

The original data can be obtained by contacting the corresponding author, subject to the corresponding author's approval.
