# Supplementary material for: Neoadjuvant chemoimmunotherapy versus chemoradiotherapy for esophageal squamous cell carcinoma: a multicenter cohort study
Source: Front Immunol. 2026 Jul 13;17:1882240. doi: 10.3389/fimmu.2026.1882240 (PMC13402408; doi:10.3389/fimmu.2026.1882240)
Supplement: Supplementary file 1 [file DataSheet1.docx]

**Supplement figures and tables**


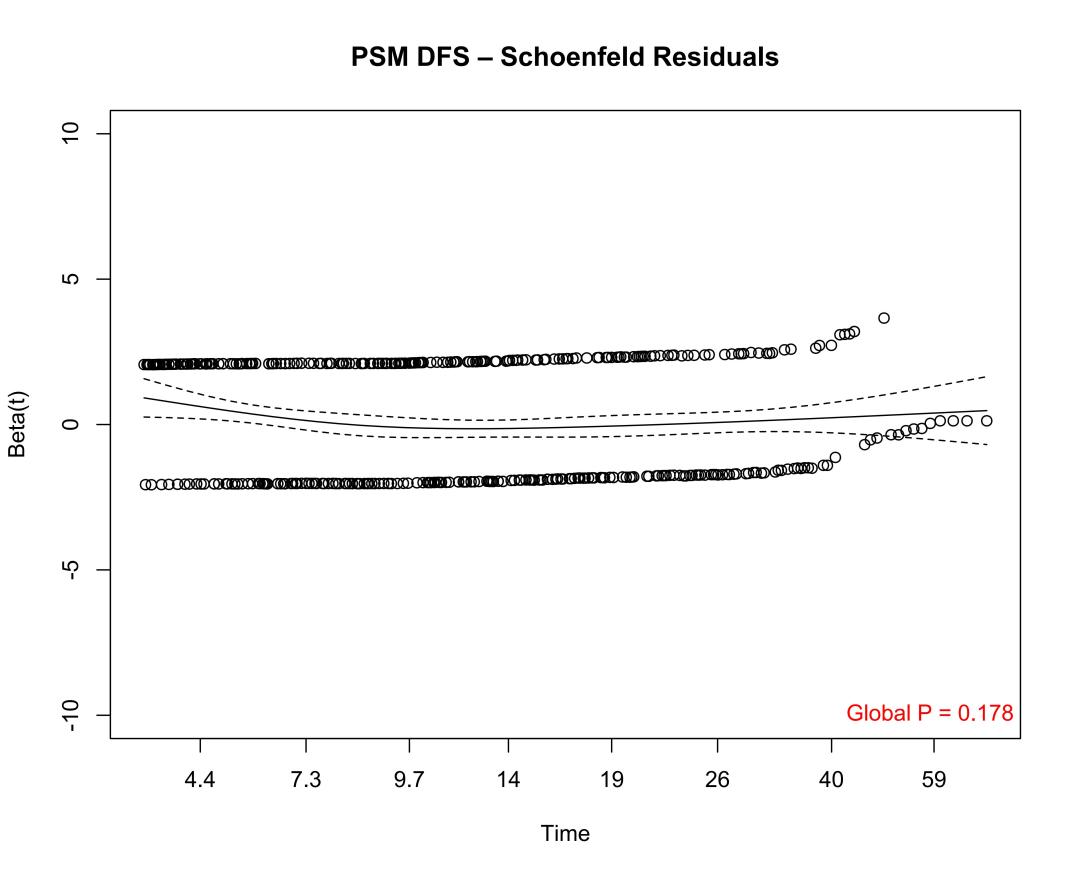
**Figure S1:** Schoenfeld residual plots for DFS after PSM. DFS, disease-free survival; PSM, propensity score matching.

The P-value for Schoenfeld residuals was >0.05, indicating that the Cox model met the proportional hazards assumption.


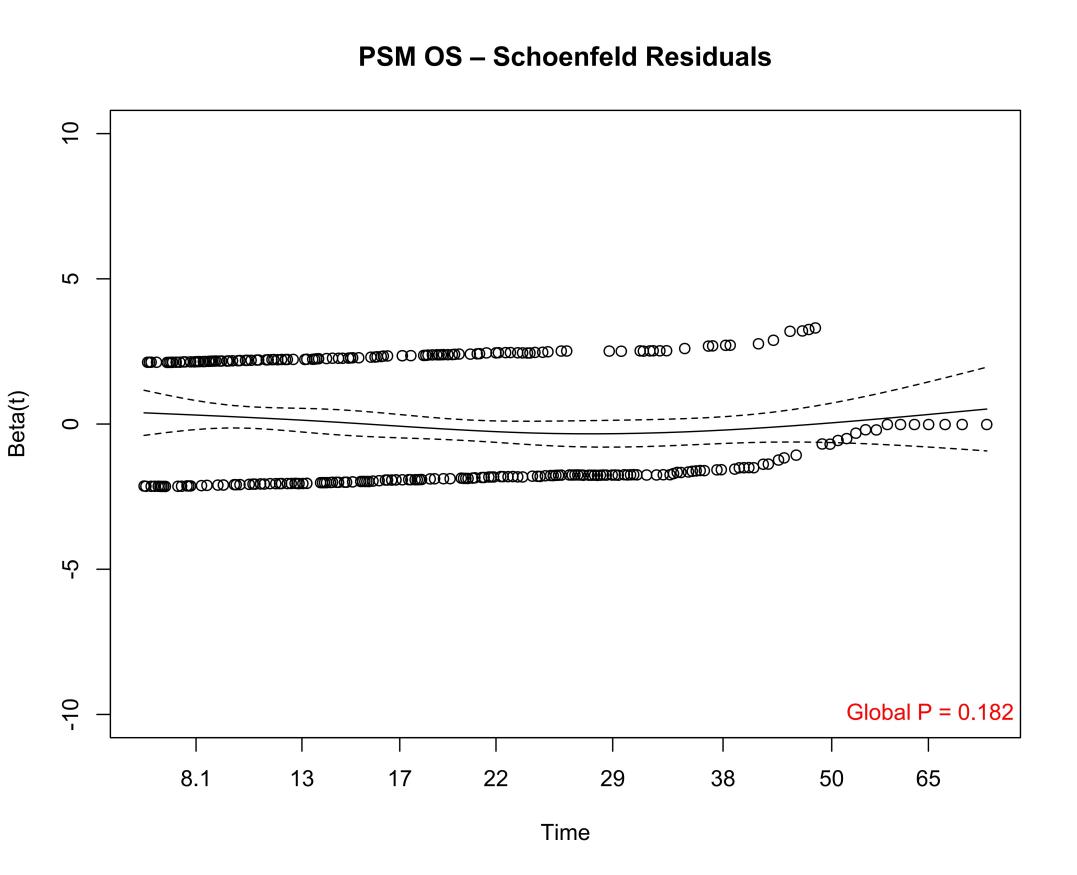


**Figure S2:** Schoenfeld residual plots for OS after PSM. OS, overall survial; PSM, propensity score matching.

The P-value for Schoenfeld residuals was >0.05, indicating that the Cox model met the proportional hazards assumption.


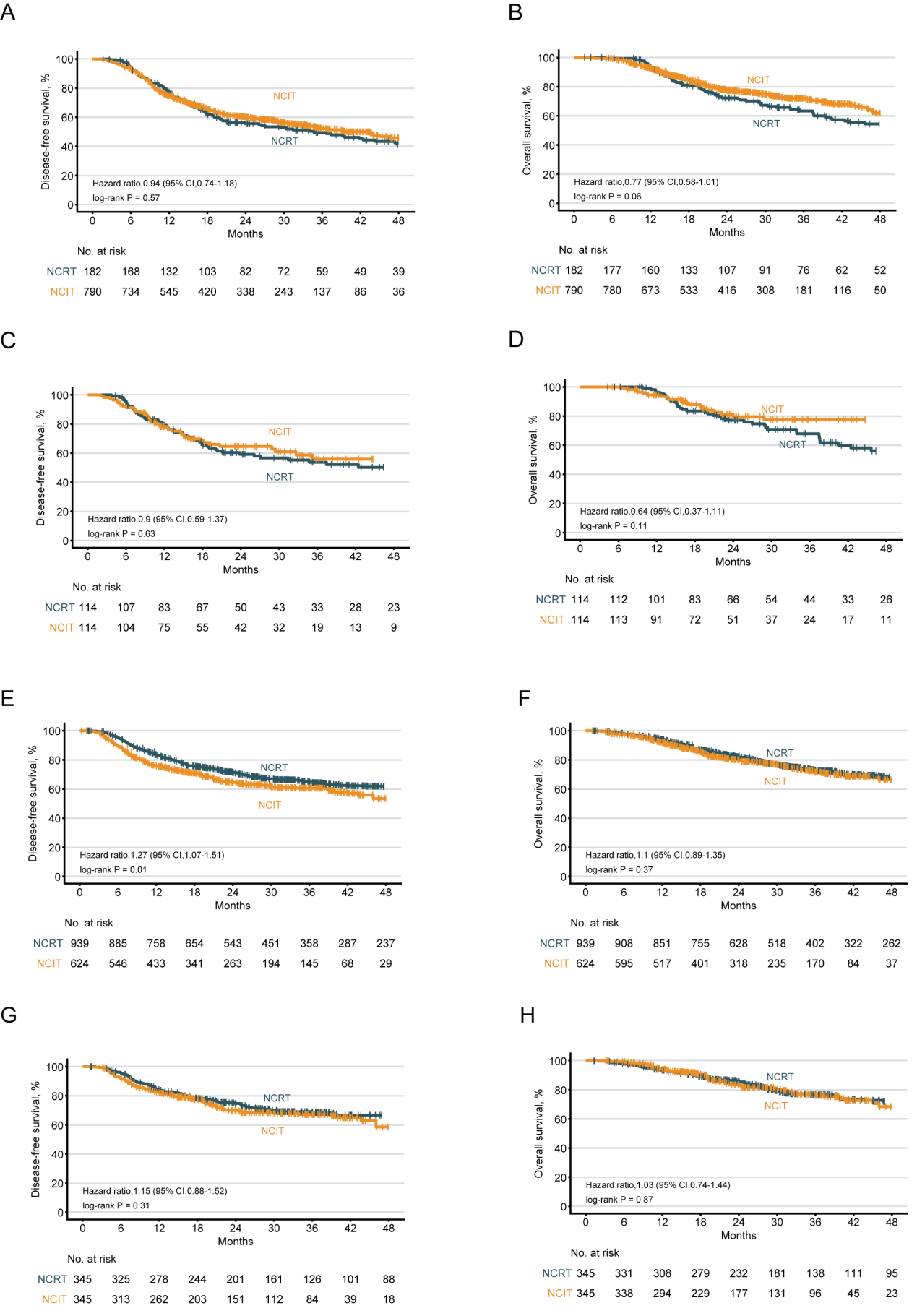


Figure S3. Kaplan-Meier curve of NCIT and NCRT in patients receiving adjuvant therapy or not.

Pre-PSM patient DFS (A) and OS (B) curves in those receiving adjuvant therapy; post-PSM patient DFS (C) and OS (D) curves in those receiving adjuvant therapy. Pre-PSM patient DFS (A) and OS (B) curves in those without adjuvant therapy; post-PSM patient DFS (C) and OS (D) curves in those without adjuvant therapy. NCIT, neoadjuvant chemotherapy plus immunotherapy; NCRT, neoadjuvant chemoradiotherapy.


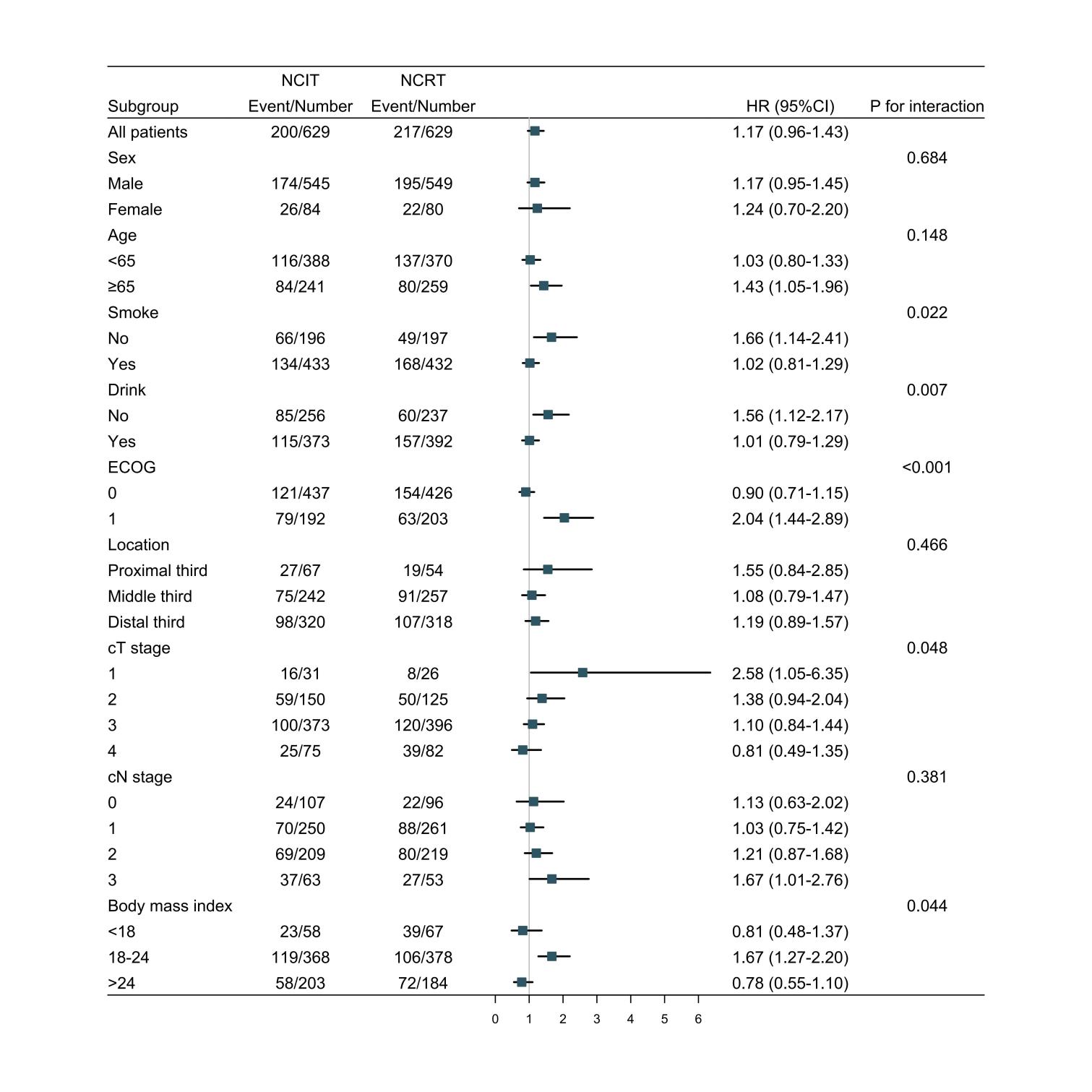
**Figure S4:** Forest plot of DFS after PSM by subgroup. DFS, disease-free survival; PSM, propensity score matching.


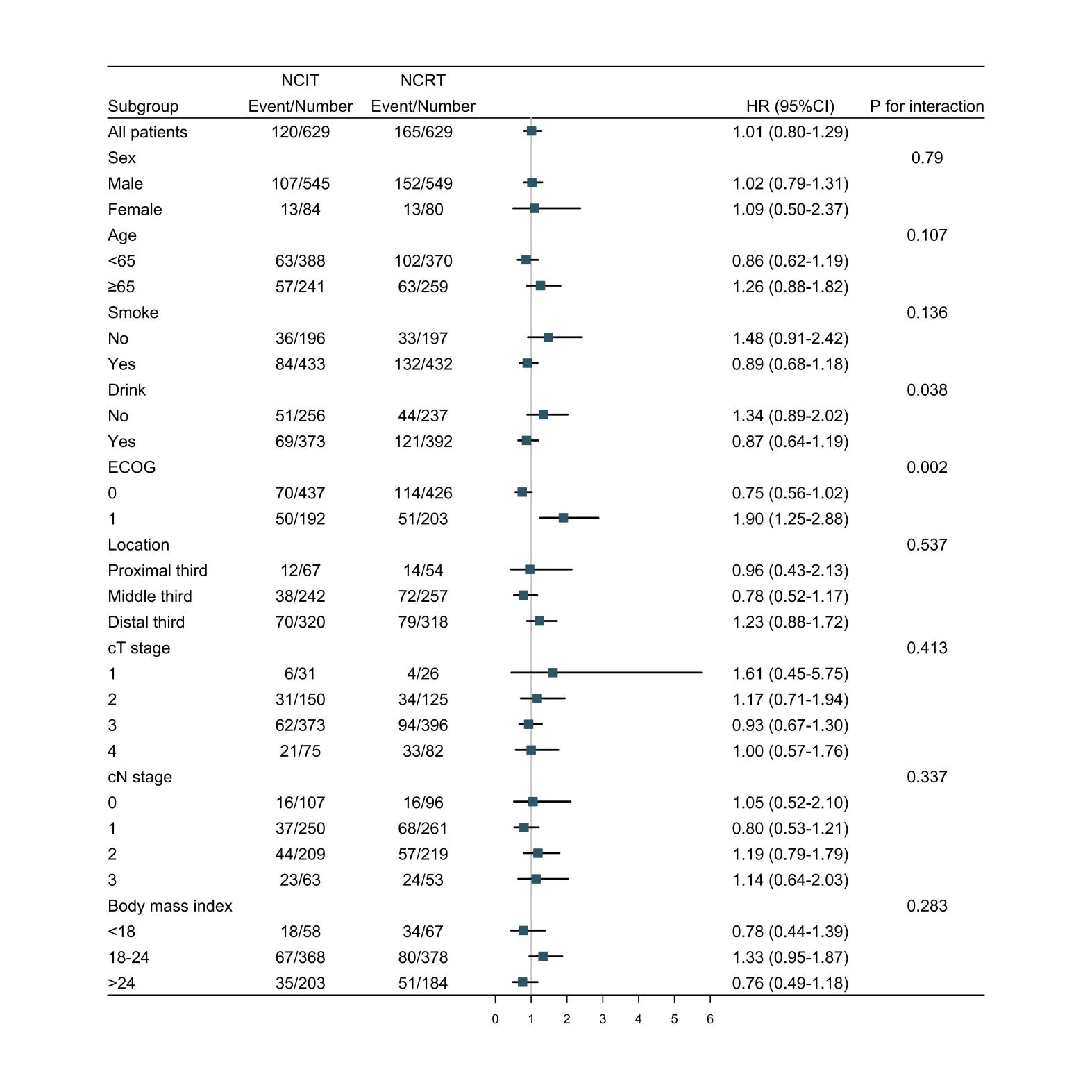
**Figure S5:** Forest plot of OS after PSM by subgroup. OS, overall survial; PSM, propensity score matching.


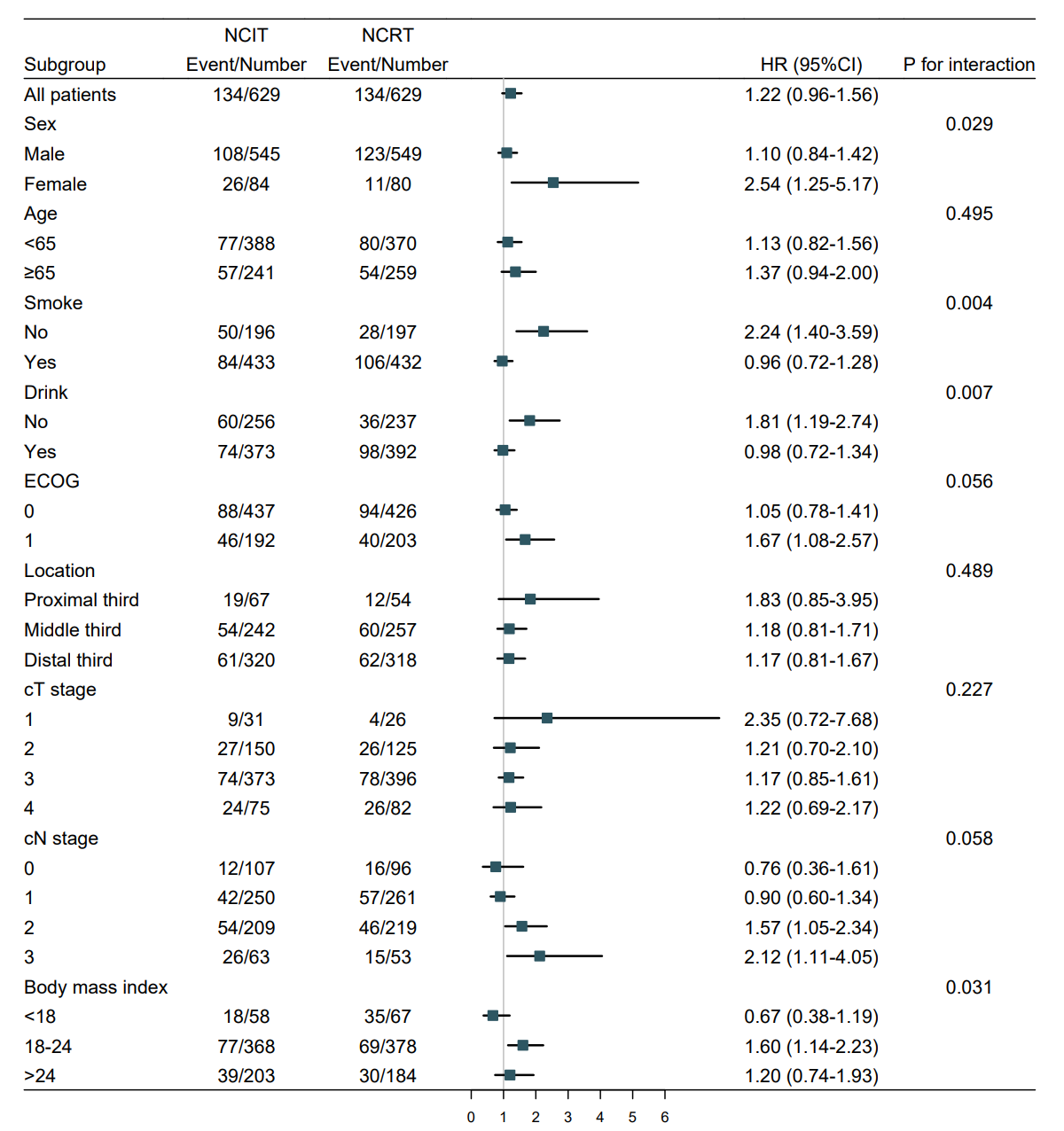


**Figure S6:** Forest plot of LRFS after PSM by subgroup. LRFS, locoregional recurrence-free survival; PSM, propensity score matching.

**
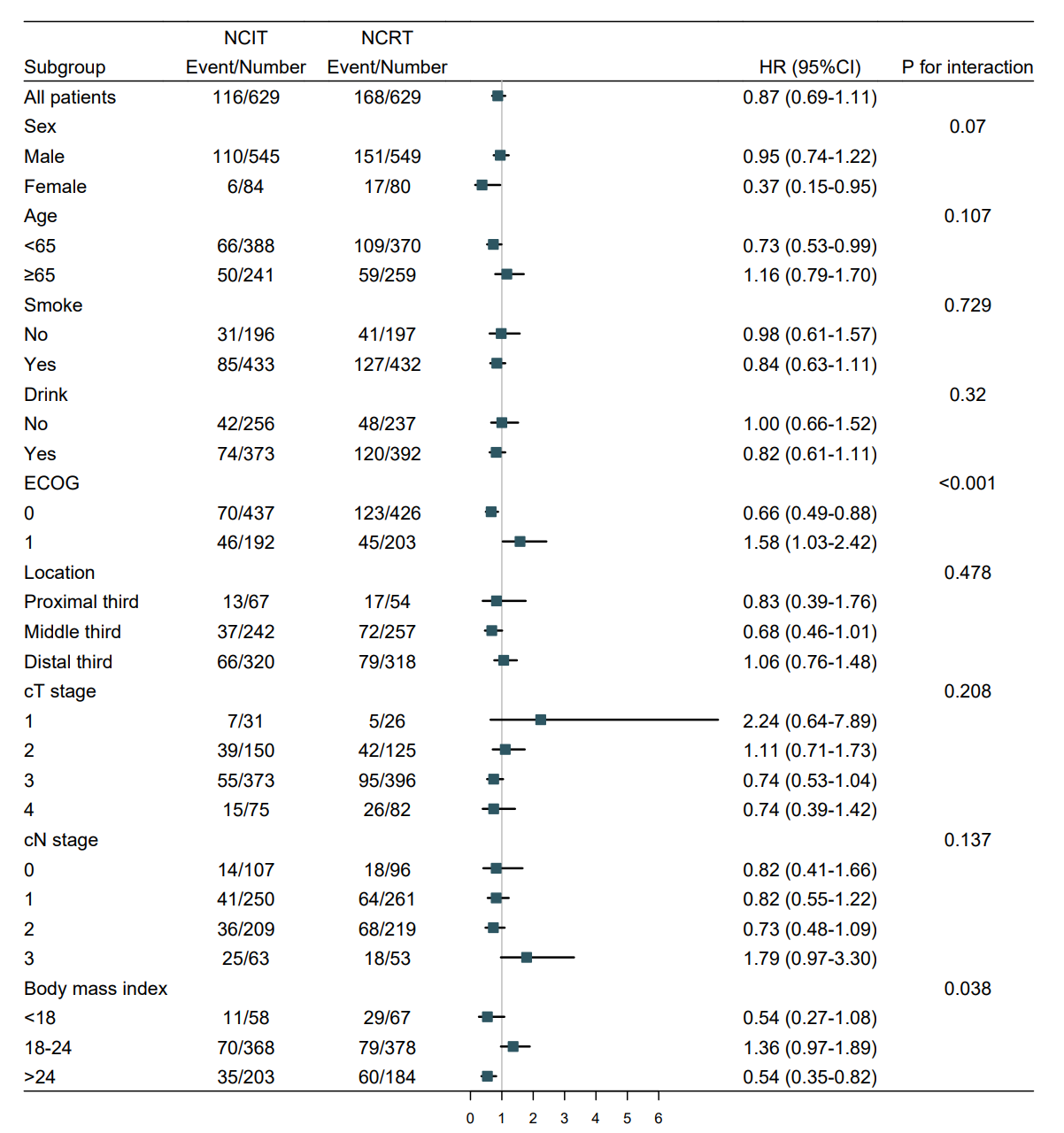
**

**Figure S7:** Forest plot of DMFS after PSM by subgroup. DMFS, distant metastasis-free survival; PSM, propensity score matching.

**Table S1.** Patient characteristics of patients with adjuvant therapy between the NCRT and NCIT groups before and after propensity score matching.

|  |  | Before propensity score matching | | | |  |  | After propensity score matching | | | |
| --- | --- | --- | --- | --- | --- | --- | --- | --- | --- | --- | --- |
| Characteristic |  | NCIT (n = 790) | NCRT (n = 182) | *P* value | SMD |  | NCIT (n = 114) | | NCRT (n = 114) | *P* value | SMD |
| Sex |  |  |  |  |  |  |  | |  |  |  |
|  | Male | 691 (87.5) | 162 (89.0) | 0.655 | 0.048 |  | 100 (87.7) | | 101 (88.6) | 1 | 0.027 |
|  | Female | 99 (12.5) | 20 ( 11.0) |  |  |  | 14 (12.3) | | 13 (11.4) |  |  |
| Age |  |  |  |  |  |  |  | |  |  |  |
|  | <65 | 469 (59.4) | 132 (72.5) | 0.001 | 0.28 |  | 77 (67.5) | | 75 (65.8) | 0.888 | 0.037 |
|  | ≥65 | 321 (40.6) | 50 (27.5) |  |  |  | 37 (32.5) | | 39 (34.2) |  |  |
| Smok |  |  |  |  |  |  |  | |  |  |  |
|  | No | 255 (32.3) | 39 (21.4) | 0.005 | 0.247 |  | 30 (26.3) | | 28 (24.6) | 0.879 | 0.04 |
|  | Yes | 535 (67.7) | 143 (78.6) |  |  |  | 84 (73.7) | | 86 (75.4) |  |  |
| Drink |  |  |  |  |  |  |  | |  |  |  |
|  | No | 318 (40.3) | 49 (26.9) | 0.001 | 0.285 |  | 36 (31.6) | | 37 (32.5) | 1 | 0.019 |
|  | Yes | 472 (59.7) | 133 (73.1) |  |  |  | 78 (68.4) | | 77 (67.5) |  |  |
| ECOG |  |  |  |  |  |  |  | |  |  |  |
|  | 0 | 606 (76.7) | 61 (33.5) | <0.001 | 0.964 |  | 54 (47.4) | | 57 (50.0) | 0.791 | 0.053 |
|  | 1 | 184 (23.3) | 121 (66.5) |  |  |  | 60 (52.6) | | 57 (50.0) |  |  |
| cT stage |  |  |  |  |  |  |  | |  |  |  |
|  | 1 | 71 (9.0) | 4 (2.2) | <0.001 | 0.543 |  | 7 (6.1) | | 4 (3.5) | 0.828 | 0.125 |
|  | 2 | 110 (13.9) | 24 (13.2) |  |  |  | 22 (19.3) | | 22 (19.3) |  |  |
|  | 3 | 556 (70.4) | 112 (61.5) |  |  |  | 74 (64.9) | | 76 ( 66.7) |  |  |
|  | 4 | 53 (6.7) | 42 (23.1) |  |  |  | 11 (9.6) | | 12 (10.5) |  |  |
| cN stage |  |  |  |  |  |  |  | |  |  |  |
|  | 0 | 138 ( 17.5) | 20 (11.0) | 0.061 | 0.231 |  | 12 (10.5) | | 11 (9.6) | 0.977 | 0.06 |
|  | 1 | 320 ( 40.5) | 74 (40.7) |  |  |  | 40 (35.1) | | 43 (37.7) |  |  |
|  | 2 | 256 (32.4) | 74 (40.7) |  |  |  | 49 (43.0) | | 48 ( 42.1) |  |  |
|  | 3 | 76 (9.6) | 14 (7.7) |  |  |  | 13 (11.4) | | 12 (10.5) |  |  |
| Tumor location |  |  |  |  |  |  |  | |  |  |  |
|  | Proximal third | 70 (8.9) | 14 (7.7) | 0.151 | 0.16 |  | 19 (16.7) | | 8 (7.0) | 0.048 | 0.331 |
|  | Middle third | 362 (45.8) | 71 (39.0) |  |  |  | 47 (41.2) | | 45 (39.5) |  |  |
|  | Distal third | 358 (45.3) | 97 (53.3) |  |  |  | 48 (42.1) | | 61 (53.5) |  |  |
| BMI |  |  |  |  |  |  |  | |  |  |  |
|  | <18 | 40 (5.1) | 38 (20.9) | <0.001 | 0.503 |  | 8 (7.0) | | 8 (7.0) | 0.85 | 0.076 |
|  | 18-24 | 493 (62.4) | 85 (46.7) |  |  |  | 65 (57.0) | | 69 (60.5) |  |  |
|  | >24 | 257 (32.5) | 59 ( 32.4) |  |  |  | 41 (36.0) | | 37 (32.5) |  |  |
| Adjuvant therapy | Yes | 790 (100.0) | 182 (100.0) | NA | <0.001 |  | 114 (100.0) | | 114 (100.0) | NA | <0.001 |

NCIT, neoadjuvant chemotherapy plus immunotherapy; NCRT, neoadjuvant chemoradiotherapy; BMI, Body mass index.

**Table S2.** Patient characteristics of patients without adjuvant therapy between the NCRT and NCIT groups before and after propensity score matching.

|  |  | Before propensity score matching | | | |  | After propensity score matching | | | |
| --- | --- | --- | --- | --- | --- | --- | --- | --- | --- | --- |
| Characteristic |  | NCIT (n = 624) | NCRT (n = 939) | *P* value | SMD |  | NCIT (n = 345) | NCRT (n = 345) | *P* value | SMD |
| Sex |  |  |  |  |  |  |  |  |  |  |
|  | Male | 512 (82.1) | 779 (83.0) | 0.692 | 0.024 |  | 272 (78.8) | 291 (84.3) | 0.077 | 0.142 |
|  | Female | 112 (17.9) | 160 (17.0) |  |  |  | 73 (21.2) | 54 (15.7) |  |  |
| Age |  |  |  |  |  |  |  |  |  |  |
|  | <65 | 360 (57.7) | 570 (60.7) | 0.256 | 0.061 |  | 208 (60.3) | 197 (57.1) | 0.439 | 0.065 |
|  | ≥65 | 264 (42.3) | 369 (39.3) |  |  |  | 137 (39.7) | 148 (42.9) |  |  |
| Smok |  |  |  |  |  |  |  |  |  |  |
|  | No | 216 (34.6) | 319 (34.0) | 0.835 | 0.014 |  | 142 (41.2) | 121 (35.1) | 0.117 | 0.126 |
|  | Yes | 408 (65.4) | 620 (66.0) |  |  |  | 203 (58.8) | 224 (64.9) |  |  |
| Drink |  |  |  |  |  |  |  |  |  |  |
|  | No | 243 (38.9) | 343 (36.5) | 0.362 | 0.05 |  | 157 (45.5) | 136 (39.4) | 0.123 | 0.123 |
|  | Yes | 381 (61.1) | 596 (63.5) |  |  |  | 188 (54.5) | 209 (60.6) |  |  |
| ECOG |  |  |  |  |  |  |  |  |  |  |
|  | 0 | 570 (91.3) | 573 (61.0) | <0.001 | 0.762 |  | 291 (84.3) | 280 (81.2) | 0.314 | 0.084 |
|  | 1 | 54 (8.7) | 366 ( 39.0) |  |  |  | 54 (15.7) | 65 (18.8) |  |  |
| cT stage |  |  |  |  |  |  |  |  |  |  |
|  | 1 | 10 (1.6) | 24 (2.6) | <0.001 | 0.767 |  | 8 (2.3) | 9 (2.6) | 0.214 | 0.162 |
|  | 2 | 65 (10.4) | 336 (35.8) |  |  |  | 63 (18.3) | 43 (12.5) |  |  |
|  | 3 | 494 (79.2) | 425 (45.3) |  |  |  | 231 (67.0) | 248 (71.9) |  |  |
|  | 4 | 55 (8.8) | 154 (16.4) |  |  |  | 43 (12.5) | 45 (13.0) |  |  |
| cN stage |  |  |  |  |  |  |  |  |  |  |
|  | 0 | 126 (20.2) | 108 (11.5) | <0.001 | 0.262 |  | 71 (20.6) | 61 (17.7) | 0.429 | 0.127 |
|  | 1 | 268 (42.9) | 400 (42.6) |  |  |  | 143 (41.4) | 138 ( 40.0) |  |  |
|  | 2 | 195 (31.2) | 367 (39.1) |  |  |  | 109 (31.6) | 128 (37.1) |  |  |
|  | 3 | 35 (5.6) | 64 (6.8) |  |  |  | 22 (6.4) | 18 (5.2) |  |  |
| Tumor location |  |  |  |  |  |  |  |  |  |  |
|  | Proximal third | 60 (9.6) | 102 (10.9) | 0.062 | 0.122 |  | 33 (9.6) | 32 (9.3) | 0.864 | 0.041 |
|  | Middle third | 284 (45.5) | 371 (39.5) |  |  |  | 141 (40.9) | 148 (42.9) |  |  |
|  | Distal third | 280 (44.9) | 466 (49.6) |  |  |  | 171 (49.6) | 165 (47.8) |  |  |
| BMI |  |  |  |  |  |  |  |  |  |  |
|  | <18 | 32 (5.1) | 125 (13.3) | <0.001 | 0.293 |  | 26 (7.5) | 33 (9.6) | 0.543 | 0.084 |
|  | 18-24 | 407 (65.2) | 534 (56.9) |  |  |  | 208 (60.3) | 210 (60.9) |  |  |
|  | >24 | 185 (29.6) | 280 (29.8) |  |  |  | 111 (32.2) | 102 (29.6) |  |  |
| Adjuvant therapy | No | 624 (100.0) | 939 (100.0) | NA | <0.001 |  | 345 (100.0) | 345 (100.0) | NA | <0.001 |

NCIT, neoadjuvant chemotherapy plus immunotherapy; NCRT, neoadjuvant chemoradiotherapy; BMI, Body mass index.

**Table S3.** The failure pattern between patients receiving NCIT and NCRT after PSM

|  | Before PSM | | After PSM | |
| --- | --- | --- | --- | --- |
| Failure type | NCIT (n=1414) | NCRT (n=1121) | NCIT (n=629) | NCRT (n=629) |
| None | 654 (60.3) | 685 (61.1) | 429 (68.2) | 412 (65.5) |
| Local | 221 (15.6) | 95 (8.4) | 84 (13.3) | 49 (7.7) |
| Distant | 175 (12.3) | 169 (15.0) | 66 (10.5) | 83 (13.1) |
| Local and distant | 87 (6.1) | 68 (6.0) | 28 (4.5) | 36 (5.7) |
| Death | 77 (5.4) | 104 (9.2) | 22 (3.5) | 49 (7.7) |

PSM, propensity score matching; NCIT, neoadjuvant chemotherapy plus immunotherapy; NCRT, neoadjuvant chemoradiotherapy.
